# 5-Bromo-2-phenyl-3-phenyl­sulfinyl-1-benzofuran

**DOI:** 10.1107/S1600536809039324

**Published:** 2009-10-03

**Authors:** Hong Dae Choi, Pil Ja Seo, Byeng Wha Son, Uk Lee

**Affiliations:** a Department of Chemistry, Dongeui University, San 24 Kaya-dong Busanjin-gu, Busan 614-714, Republic of Korea; b Department of Chemistry, Pukyong National University, 599-1 Daeyeon 3-dong, Nam-gu, Busan 608-737, Republic of Korea

## Abstract

In the title compound, C_20_H_13_BrO_2_S, the O atom and the phenyl group of the phenyl­sulfinyl substituent are located on opposite sides of the plane of the benzofuran system. The S-bound phenyl ring is almost perpendicular to this plane [80.35 (8)°]. The phenyl ring in the 2-position is twisted with respect to the benzofuran plane, making a dihedral angle of 16.0 (1)°.

## Related literature

For the crystal structures of similar 5-halo-2-phenyl-3-phenyl­sulfinyl-1-benzofuran derivatives, see: Choi *et al.* (2009*a* ▶,*b* ▶). For the pharmacological activity of benzofuran compounds, see: Howlett *et al.* (1999 ▶); Twyman & Allsop (1999 ▶). For natural products involving a benzofuran ring system, see: Akgul & Anil (2003 ▶); Reuss & König (2004 ▶).
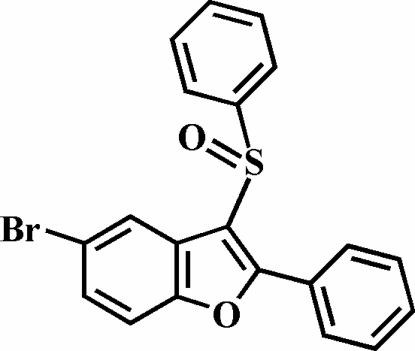


## Experimental

### 

#### Crystal data

C_20_H_13_BrO_2_S
*M* *_r_* = 397.27
Triclinic, 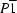

*a* = 8.2670 (1) Å
*b* = 9.5233 (2) Å
*c* = 11.8663 (2) Å
α = 72.187 (1)°
β = 80.772 (1)°
γ = 69.526 (1)°
*V* = 831.76 (2) Å^3^
*Z* = 2
Mo *K*α radiation
μ = 2.61 mm^−1^
*T* = 293 K
0.35 × 0.22 × 0.11 mm

#### Data collection

Bruker SMART CCD diffractometer
Absorption correction: multi-scan (*SADABS*; Sheldrick, 2000 ▶) *T*_min_ = 0.462, *T*_max_ = 0.763
13542 measured reflections
3275 independent reflections
2716 reflections with *I* > 2σ(*I*)
*R*_int_ = 0.030

#### Refinement

*R*[*F* ^2^ > 2σ(*F* ^2^)] = 0.035
*wR*(*F* ^2^) = 0.095
*S* = 1.05
3275 reflections
217 parameters
H-atom parameters constrained
Δρ_max_ = 0.51 e Å^−3^
Δρ_min_ = −0.59 e Å^−3^

### 

Data collection: *SMART* (Bruker, 2001 ▶); cell refinement: *SAINT* (Bruker, 2001 ▶); data reduction: *SAINT*; program(s) used to solve structure: *SHELXS97* (Sheldrick, 2008 ▶); program(s) used to refine structure: *SHELXL97* (Sheldrick, 2008 ▶); molecular graphics: *ORTEPIII* (Burnett & Johnson, 1996 ▶) and *ORTEP-3 for Windows* (Farrugia, 1997 ▶); software used to prepare material for publication: *SHELXL97*.

## Supplementary Material

Crystal structure: contains datablocks global, I. DOI: 10.1107/S1600536809039324/dn2491sup1.cif

Structure factors: contains datablocks I. DOI: 10.1107/S1600536809039324/dn2491Isup2.hkl

Additional supplementary materials:  crystallographic information; 3D view; checkCIF report

## Figures and Tables

**Fig. 1.**
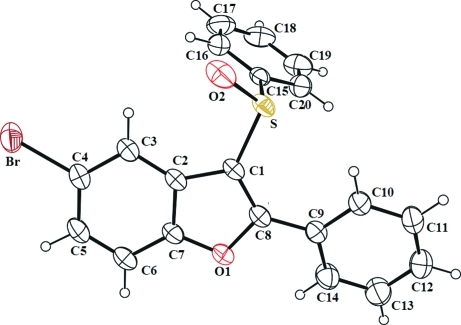
The molecular structure of the title compound with the atom numbering scheme. Displacement ellipsoids are drawn at the 30% probability level. H atoms are presented as small spheres of arbitrary radii.

## supplementary crystallographic information

### Comment

Molecules containing benzofuran moiety have received much attenttion in the field of their pharmacological properties (Howlett *et al.*, 1999; Twyman & Allsop, 1999), and these compounds are ubiquitous in nature (Akgul & Anil, 2003; Reuss & König, 2004). As a part of our ongoing studies of the effect of side chain substituents on the solid state structures of 5-halo-2-phenyl-3-phenylsulfinyl-1-benzofuran analogues (Choi *et al.*, 2009*a,b*), we present the crystal structure of the title compound (Fig. 1).

The benzofuran unit is essentially planar, with a mean deviation of 0.010 (2) Å from the least-squares plane defined by the nine constituent atoms. The dihedral angle formed by the plane of the benzofuran unit and the plane of 2-phenyl ring is 16.0 (1)°. The phenyl ring (C15-C20) is almost perpendicular to the plane of the benzofuran unit [80.35 (8)°].

### Experimental

77% 3-Chloroperoxybenzoic acid (224 mg, 1.0 mmol) was added in small portions to a stirred solution of 5-bromo-2-phenyl-3-phenylsulfanyl-1-benzofuran (343 mg, 0.9 mmol) in dichloromethane (30 mL) at 273 K. After being stirred at room temperature for 4h, the mixture was washed with saturated sodium bicarbonate solution and the organic layer was separated, dried over magnesium sulfate, filtered and concentrated in vacuum. The residue was purified by column chromatography (hexane-ethyl acetate, 2 : 1 v/v) to afford the title compound as a colorless solid [yield 73%, m.p. 417-418 K; *R*_f_ = 0.56 (hexane-ethyl acetate, 2 : 1 v/v)]. Single crystals suitable for X-ray diffraction were prepared by slow evaporation of a solution of the title compound in benzene at room temperature.

### Refinement

All H atoms were fixed geometrically and treated as riding with C–H = 0.93 Å and U_iso_(H) = 1.2U_eq_(C)

### Crystal data

**Table:**

C_20_H_13_BrO_2_S	*Z* = 2
*M**_r_* = 397.27	*F*(000) = 400
Triclinic, *P*1	*D*_x_ = 1.586 Mg m^−^^3^
Hall symbol: -P 1	Mo *K*α radiation, λ = 0.71073 Å
*a* = 8.2670 (1) Å	Cell parameters from 5885 reflections
*b* = 9.5233 (2) Å	θ = 2.4–26.0°
*c* = 11.8663 (2) Å	µ = 2.61 mm^−^^1^
α = 72.187 (1)°	*T* = 293 K
β = 80.772 (1)°	Block, colorless
γ = 69.526 (1)°	0.35 × 0.22 × 0.11 mm
*V* = 831.76 (2) Å^3^	

### Data collection

**Table:**

Bruker SMART CCD diffractometer	3275 independent reflections
Radiation source: fine-focus sealed tube	2716 reflections with *I* > 2σ(*I*)
graphite	*R*_int_ = 0.030
Detector resolution: 10.0 pixels mm^-1^	θ_max_ = 26.0°, θ_min_ = 1.8°
φ and ω scans	*h* = −10→10
Absorption correction: multi-scan (*SADABS*; Sheldrick, 2000)	*k* = −11→11
*T*_min_ = 0.462, *T*_max_ = 0.763	*l* = −14→14
13542 measured reflections	

### Refinement

**Table:**

Refinement on *F*^2^	Primary atom site location: structure-invariant direct methods
Least-squares matrix: full	Secondary atom site location: difference Fourier map
*R*[*F*^2^ > 2σ(*F*^2^)] = 0.035	Hydrogen site location: difference Fourier map
*wR*(*F*^2^) = 0.095	H-atom parameters constrained
*S* = 1.05	*w* = 1/[σ^2^(*F*_o_^2^) + (0.0473*P*)^2^ + 0.3341*P*] where *P* = (*F*_o_^2^ + 2*F*_c_^2^)/3
3275 reflections	(Δ/σ)_max_ = 0.001
217 parameters	Δρ_max_ = 0.51 e Å^−^^3^
0 restraints	Δρ_min_ = −0.59 e Å^−^^3^

### Special details

**Table:**

Geometry. All esds (except the esd in the dihedral angle between two l.s. planes) are estimated using the full covariance matrix. The cell esds are taken into account individually in the estimation of esds in distances, angles and torsion angles; correlations between esds in cell parameters are only used when they are defined by crystal symmetry. An approximate (isotropic) treatment of cell esds is used for estimating esds involving l.s. planes.
Refinement. Refinement of F^2^ against ALL reflections. The weighted R-factor wR and goodness of fit S are based on F^2^, conventional R-factors R are based on F, with F set to zero for negative F^2^. The threshold expression of F^2^ > 2sigma(F^2^) is used only for calculating R-factors(gt) etc. and is not relevant to the choice of reflections for refinement. R-factors based on F^2^ are statistically about twice as large as those based on F, and R- factors based on ALL data will be even larger.

### Fractional atomic coordinates and isotropic or equivalent isotropic displacement parameters (Å^2^)

**Table:**

	*x*	*y*	*z*	*U*_iso_*/*U*_eq_	
Br	1.01046 (4)	0.24078 (4)	0.38069 (3)	0.07875 (15)	
S	0.60607 (8)	0.10785 (7)	0.86253 (6)	0.05063 (17)	
O1	0.5011 (2)	0.56165 (18)	0.71087 (15)	0.0552 (4)	
O2	0.7801 (2)	0.0093 (2)	0.8283 (2)	0.0760 (6)	
C1	0.5823 (3)	0.3012 (2)	0.7751 (2)	0.0453 (5)	
C2	0.6714 (3)	0.3415 (3)	0.6609 (2)	0.0470 (5)	
C3	0.7888 (3)	0.2607 (3)	0.5871 (2)	0.0516 (6)	
H3	0.8269	0.1525	0.6070	0.062*	
C4	0.8466 (3)	0.3472 (3)	0.4831 (2)	0.0566 (6)	
C5	0.7910 (4)	0.5094 (3)	0.4506 (3)	0.0655 (7)	
H5	0.8338	0.5632	0.3796	0.079*	
C6	0.6738 (4)	0.5895 (3)	0.5225 (3)	0.0645 (7)	
H6	0.6343	0.6978	0.5017	0.077*	
C7	0.6166 (3)	0.5035 (3)	0.6272 (2)	0.0518 (6)	
C8	0.4830 (3)	0.4365 (3)	0.8020 (2)	0.0486 (5)	
C9	0.3645 (3)	0.4760 (3)	0.9012 (2)	0.0505 (6)	
C10	0.3612 (4)	0.3686 (3)	1.0094 (3)	0.0660 (7)	
H10	0.4365	0.2672	1.0207	0.079*	
C11	0.2474 (4)	0.4101 (4)	1.1009 (3)	0.0750 (8)	
H11	0.2469	0.3363	1.1735	0.090*	
C12	0.1352 (4)	0.5581 (4)	1.0866 (3)	0.0768 (9)	
H12	0.0581	0.5850	1.1487	0.092*	
C13	0.1375 (5)	0.6667 (4)	0.9799 (3)	0.0868 (10)	
H13	0.0622	0.7680	0.9696	0.104*	
C14	0.2507 (4)	0.6265 (3)	0.8877 (3)	0.0732 (8)	
H14	0.2511	0.7011	0.8156	0.088*	
C15	0.4515 (3)	0.0742 (2)	0.7931 (2)	0.0455 (5)	
C16	0.5017 (4)	0.0001 (3)	0.7038 (3)	0.0647 (7)	
H16	0.6170	−0.0307	0.6762	0.078*	
C17	0.3767 (6)	−0.0275 (4)	0.6558 (3)	0.0833 (10)	
H17	0.4082	−0.0778	0.5959	0.100*	
C18	0.2085 (5)	0.0188 (4)	0.6965 (4)	0.0836 (10)	
H18	0.1254	0.0015	0.6629	0.100*	
C19	0.1600 (4)	0.0901 (4)	0.7856 (4)	0.0791 (10)	
H19	0.0445	0.1200	0.8129	0.095*	
C20	0.2804 (3)	0.1180 (3)	0.8351 (3)	0.0592 (6)	
H20	0.2474	0.1659	0.8964	0.071*	

### Atomic displacement parameters (Å^2^)

**Table:**

	*U*^11^	*U*^22^	*U*^33^	*U*^12^	*U*^13^	*U*^23^
Br	0.0790 (2)	0.0939 (3)	0.0630 (2)	−0.03538 (18)	0.01864 (16)	−0.02325 (17)
S	0.0434 (3)	0.0442 (3)	0.0520 (4)	−0.0148 (2)	−0.0066 (3)	0.0070 (2)
O1	0.0669 (11)	0.0419 (8)	0.0506 (10)	−0.0212 (8)	−0.0008 (8)	−0.0010 (7)
O2	0.0417 (10)	0.0554 (10)	0.1019 (16)	−0.0060 (8)	−0.0054 (10)	0.0088 (10)
C1	0.0446 (12)	0.0424 (11)	0.0450 (13)	−0.0191 (9)	−0.0066 (10)	0.0017 (9)
C2	0.0452 (12)	0.0475 (12)	0.0462 (13)	−0.0220 (10)	−0.0059 (10)	0.0002 (10)
C3	0.0468 (13)	0.0515 (13)	0.0524 (14)	−0.0201 (10)	−0.0030 (11)	−0.0034 (11)
C4	0.0540 (14)	0.0659 (15)	0.0496 (15)	−0.0267 (12)	0.0013 (11)	−0.0085 (12)
C5	0.0774 (19)	0.0670 (17)	0.0487 (15)	−0.0372 (15)	0.0034 (13)	0.0020 (13)
C6	0.0787 (19)	0.0507 (14)	0.0568 (16)	−0.0289 (13)	−0.0025 (14)	0.0045 (12)
C7	0.0548 (14)	0.0484 (12)	0.0489 (14)	−0.0222 (11)	−0.0026 (11)	−0.0022 (10)
C8	0.0516 (13)	0.0473 (12)	0.0438 (13)	−0.0214 (10)	−0.0071 (10)	0.0008 (10)
C9	0.0501 (13)	0.0527 (13)	0.0490 (14)	−0.0208 (11)	−0.0042 (11)	−0.0085 (11)
C10	0.0787 (19)	0.0550 (15)	0.0548 (16)	−0.0188 (13)	0.0048 (14)	−0.0090 (12)
C11	0.088 (2)	0.0731 (19)	0.0537 (17)	−0.0271 (17)	0.0110 (15)	−0.0100 (14)
C12	0.0691 (19)	0.088 (2)	0.070 (2)	−0.0255 (17)	0.0152 (15)	−0.0269 (17)
C13	0.077 (2)	0.0691 (19)	0.089 (3)	−0.0030 (16)	0.0095 (18)	−0.0164 (18)
C14	0.0709 (19)	0.0611 (16)	0.0660 (19)	−0.0098 (14)	0.0026 (15)	−0.0036 (14)
C15	0.0445 (12)	0.0335 (10)	0.0491 (13)	−0.0121 (9)	−0.0008 (10)	0.0006 (9)
C16	0.0687 (17)	0.0482 (13)	0.0679 (18)	−0.0157 (12)	0.0089 (14)	−0.0125 (12)
C17	0.127 (3)	0.0537 (16)	0.078 (2)	−0.0311 (19)	−0.018 (2)	−0.0209 (15)
C18	0.090 (3)	0.0569 (17)	0.112 (3)	−0.0276 (17)	−0.037 (2)	−0.0142 (18)
C19	0.0525 (17)	0.0681 (18)	0.116 (3)	−0.0219 (14)	−0.0125 (17)	−0.0174 (19)
C20	0.0474 (14)	0.0579 (14)	0.0708 (18)	−0.0168 (11)	0.0004 (12)	−0.0174 (13)

### Geometric parameters (Å, °)

**Table:**

Br—C4	1.899 (3)	C10—C11	1.377 (4)
S—O2	1.489 (2)	C10—H10	0.9300
S—C1	1.775 (2)	C11—C12	1.366 (5)
S—C15	1.789 (2)	C11—H11	0.9300
O1—C7	1.366 (3)	C12—C13	1.371 (5)
O1—C8	1.377 (3)	C12—H12	0.9300
C1—C8	1.365 (4)	C13—C14	1.377 (5)
C1—C2	1.444 (3)	C13—H13	0.9300
C2—C3	1.385 (4)	C14—H14	0.9300
C2—C7	1.391 (3)	C15—C16	1.379 (4)
C3—C4	1.376 (3)	C15—C20	1.383 (3)
C3—H3	0.9300	C16—C17	1.390 (5)
C4—C5	1.393 (4)	C16—H16	0.9300
C5—C6	1.366 (4)	C17—C18	1.359 (5)
C5—H5	0.9300	C17—H17	0.9300
C6—C7	1.378 (4)	C18—C19	1.361 (5)
C6—H6	0.9300	C18—H18	0.9300
C8—C9	1.459 (4)	C19—C20	1.370 (4)
C9—C10	1.379 (4)	C19—H19	0.9300
C9—C14	1.389 (4)	C20—H20	0.9300
			
O2—S—C1	106.63 (11)	C11—C10—H10	119.7
O2—S—C15	106.87 (13)	C9—C10—H10	119.7
C1—S—C15	97.41 (10)	C12—C11—C10	120.9 (3)
C7—O1—C8	107.23 (18)	C12—C11—H11	119.6
C8—C1—C2	107.74 (19)	C10—C11—H11	119.6
C8—C1—S	127.74 (19)	C11—C12—C13	119.3 (3)
C2—C1—S	124.50 (18)	C11—C12—H12	120.3
C3—C2—C7	119.5 (2)	C13—C12—H12	120.3
C3—C2—C1	136.0 (2)	C12—C13—C14	120.3 (3)
C7—C2—C1	104.5 (2)	C12—C13—H13	119.9
C4—C3—C2	117.2 (2)	C14—C13—H13	119.9
C4—C3—H3	121.4	C13—C14—C9	120.8 (3)
C2—C3—H3	121.4	C13—C14—H14	119.6
C3—C4—C5	122.7 (3)	C9—C14—H14	119.6
C3—C4—Br	118.6 (2)	C16—C15—C20	120.5 (3)
C5—C4—Br	118.8 (2)	C16—C15—S	121.3 (2)
C6—C5—C4	120.3 (2)	C20—C15—S	118.1 (2)
C6—C5—H5	119.9	C15—C16—C17	118.8 (3)
C4—C5—H5	119.9	C15—C16—H16	120.6
C5—C6—C7	117.3 (2)	C17—C16—H16	120.6
C5—C6—H6	121.4	C18—C17—C16	120.1 (3)
C7—C6—H6	121.4	C18—C17—H17	120.0
O1—C7—C6	126.1 (2)	C16—C17—H17	120.0
O1—C7—C2	110.8 (2)	C17—C18—C19	120.9 (3)
C6—C7—C2	123.0 (3)	C17—C18—H18	119.5
C1—C8—O1	109.7 (2)	C19—C18—H18	119.5
C1—C8—C9	135.1 (2)	C18—C19—C20	120.3 (3)
O1—C8—C9	115.3 (2)	C18—C19—H19	119.8
C10—C9—C14	118.1 (3)	C20—C19—H19	119.8
C10—C9—C8	122.2 (2)	C19—C20—C15	119.3 (3)
C14—C9—C8	119.6 (2)	C19—C20—H20	120.3
C11—C10—C9	120.6 (3)	C15—C20—H20	120.3
			
O2—S—C1—C8	−152.9 (2)	C7—O1—C8—C1	−1.4 (3)
C15—S—C1—C8	96.9 (2)	C7—O1—C8—C9	179.5 (2)
O2—S—C1—C2	25.7 (2)	C1—C8—C9—C10	16.4 (5)
C15—S—C1—C2	−84.4 (2)	O1—C8—C9—C10	−164.9 (2)
C8—C1—C2—C3	179.1 (3)	C1—C8—C9—C14	−163.9 (3)
S—C1—C2—C3	0.2 (4)	O1—C8—C9—C14	14.8 (4)
C8—C1—C2—C7	0.1 (3)	C14—C9—C10—C11	0.3 (5)
S—C1—C2—C7	−178.80 (18)	C8—C9—C10—C11	−180.0 (3)
C7—C2—C3—C4	0.5 (4)	C9—C10—C11—C12	0.1 (5)
C1—C2—C3—C4	−178.4 (3)	C10—C11—C12—C13	−0.6 (6)
C2—C3—C4—C5	−0.4 (4)	C11—C12—C13—C14	0.6 (6)
C2—C3—C4—Br	179.02 (18)	C12—C13—C14—C9	−0.1 (6)
C3—C4—C5—C6	−0.3 (4)	C10—C9—C14—C13	−0.3 (5)
Br—C4—C5—C6	−179.7 (2)	C8—C9—C14—C13	179.9 (3)
C4—C5—C6—C7	0.8 (4)	O2—S—C15—C16	−14.1 (2)
C8—O1—C7—C6	−178.7 (3)	C1—S—C15—C16	95.8 (2)
C8—O1—C7—C2	1.5 (3)	O2—S—C15—C20	162.89 (18)
C5—C6—C7—O1	179.6 (3)	C1—S—C15—C20	−87.2 (2)
C5—C6—C7—C2	−0.7 (4)	C20—C15—C16—C17	0.9 (4)
C3—C2—C7—O1	179.8 (2)	S—C15—C16—C17	177.9 (2)
C1—C2—C7—O1	−1.0 (3)	C15—C16—C17—C18	0.3 (4)
C3—C2—C7—C6	0.0 (4)	C16—C17—C18—C19	−1.2 (5)
C1—C2—C7—C6	179.2 (3)	C17—C18—C19—C20	0.7 (5)
C2—C1—C8—O1	0.9 (3)	C18—C19—C20—C15	0.5 (5)
S—C1—C8—O1	179.67 (17)	C16—C15—C20—C19	−1.3 (4)
C2—C1—C8—C9	179.6 (3)	S—C15—C20—C19	−178.4 (2)
S—C1—C8—C9	−1.6 (4)		
